# How paediatric HIV services weathered the COVID-19 storm in Tshwane District, South Africa

**DOI:** 10.4102/sajhivmed.v25i1.1557

**Published:** 2024-05-20

**Authors:** Michael Christie, Ahmad Haeri Mazanderani, Gayle Sherman, Ute Feucht

**Affiliations:** 1 Department of Paediatrics and Child Health, Faculty of Health Sciences, University of Pretoria, Pretoria, South Africa; 2 Research Centre for Maternal, Fetal, Newborn and Child Health Care Strategies, Faculty of Health Sciences, University of Pretoria, Pretoria, South Africa; 3 Research Unit for Maternal and Infant Health Care Strategies, South African Medical Research Council, Pretoria, South Africa; 4 Centre for HIV and STIs, National Institute for Communicable Diseases, National Health Laboratory Service, Johannesburg, South Africa; 5 Department of Paediatrics and Child Health, Faculty of Health Sciences, University of the Witwatersrand, Johannesburg, South Africa

**Keywords:** COVID-19, paediatric HIV, public health, HIV management, children living with HIV, HIV services

## Abstract

**Background:**

The coronavirus disease 2019 (COVID-19) pandemic disrupted paediatric HIV services across South Africa. Shortly before COVID-19, updated national HIV guidelines were released.

**Objectives:**

This study describes COVID-19’s impact on paediatric HIV services in Tshwane District, South Africa.

**Method:**

A retrospective review of National Institute for Communicable Diseases and District Health Information System data for Tshwane District from April 2019 to March 2022. Data included: Early Infant Diagnosis (EID), HIV viral load (VL) and CD4 monitoring and HIV management among children (< 15 years) living with HIV (CLHIV). Pre-pandemic (2019/2020) and pandemic periods (2020/2021, 2021/2022) were compared.

**Results:**

Year-on-year, HIV testing improved at 10 weeks, 6 months, and 18 months, whereas birth testing decreased. HIV EID case rates were 485 (2019/2020), 410 (2020/2021) and 454 (2021/2022). HIV EID test positivity was 0.77% – 1.2%. Antiretroviral treatment initiation declined from 2019/2020 to 2020/2021, but improved in 2021/2022.

Initial HIV VL and CD4 testing declined, with HIV VL testing increasing in 2021/2022, and CD4 testing further declining. HIV VL suppression rate among CLHIV ranged from 69% to 73%.

**Conclusion:**

Initially, COVID-19 resulted in reduced paediatric HIV services as children disengaged from care. Indicators eventually recovered to proximate pre-pandemic levels; however, compensatory increases did not occur. Thus, some children may not have returned to care.

**What this study adds:** This study shows how paediatric HIV care was affected over the entire COVID-19 pandemic period.

## Introduction

The coronavirus disease 2019 (COVID-19) pandemic period profoundly impacted lives and livelihoods: it hampered economic growth, heightened societal tensions, impacted population migration, and diverted and diminished healthcare access and resources.^1,2^ The most vulnerable population groups were disproportionately affected, including children and people living with HIV.

In 2019, just prior to the COVID-19 pandemic, 340 000 South African children (< 15 years) were living with HIV (CLHIV), with an estimated 4100 HIV-associated deaths for the year in the same age group.^3^ Although the Vertical Transmission Prevention (VTP) programme had made strides to reduce the vertical transmission rate, from 16% in 2010 to 3% in 2019, paediatric HIV prevalence remained high, predominantly due to South Africa’s high maternal antenatal HIV prevalence.^3^ In 2014, the Joint United Nations Program on HIV/AIDS (UNAIDS) set the targets of 90% of people knowing their HIV status, 90% of those knowingly living with HIV being on antiretroviral therapy (ART), and of those, 90% being virally suppressed, all by 2020 (often reported as ‘90-90-90’).^4^ By 2019, some progress had been made among South African women living with HIV (WLHIV), achieving 94-75-69; the paediatric HIV programme had not fared as well, only achieving 79-47-34 for the same year.^3^ In November 2019, the National Department of Health (NDoH) published updated HIV management guidelines for adults and children, which included changes to the infant HIV testing schedule, cessation of regular CD4 count monitoring in the context of viral suppression (HIV viral load [VL] < 1000 copies/mL) and implementation of new dolutegravir-based ART formulation for all eligible adults, adolescents and children ≥ 10 years and weighing ≥ 35 kg.^5,6^

Children were mostly spared the direct effects of the COVID-19 pandemic, the majority being asymptomatic or experiencing mild to moderate disease.^7,8,9,10,11,12,13^ The pandemic did, however, exacerbate their already precarious position within society by worsening poverty and food insecurity, increasing exposure to maltreatment and disrupting education and healthcare services.^11,12,14^ Children living in South Africa were not exempt from these challenges. The Expanded Program on Immunisation (EPI) was disrupted, with a resultant national measles outbreak arising two and half years later.^15,16,17,18^ Healthcare visitations declined because of disrupted transport services, lengthy COVID-19 screening processes, fear of infection, and constrained resources.^19,20,21^ Prior to the pandemic, the 2019 EPI national coverage survey reported that only 76.1% children received all 14 immunisation doses from birth to 18 months, failing to reach the target of 91%.^22^

In February 2020 and March 2020, the rate of COVID-19 infections began to increase across South Africa, with the government declaring a National State of Disaster on 15 March 2020, and a nationwide lockdown was implemented on 27 March 2020.^23^

## Research methods and design

### Study aim

To describe the effects of the COVID-19 pandemic on the paediatric HIV prevention, testing and treatment programme, as provided by public health facilities within Tshwane District in the Gauteng province of South Africa.

### Setting

Tshwane District has nine public sector hospitals and 74 community health centres and clinics, which serve the approximately 3.2 million residents.^24^ During 2021 there were an estimated 9000 CLHIV in the district, of whom 82% were aware of their status and 53% on ART.^25,26^

### Study period

The study period consisted of two intervals: pre-pandemic (2019/2020) and pandemic (2020/2021; 2021/2022) and aligned to the NDoH’s financial year (FY) (from April to March of the next year). During the pandemic, South Africa cycled through five risk-adjusted lockdown levels based on COVID-19 epidemiological trends and the health system’s capacity to respond to the disease burden (Online Appendix 1, Figure S1). Healthcare facilities were designated as essential services and were expected to remain operational throughout.^23,27^

### Study design

As part of the Tshwane Maternal-Child COVID-19 study, a retrospective review of the District Health Information System (DHIS) and National Institute for Communicable Diseases (NICD) Surveillance Data Warehouse (DW) public sector data was conducted. As the HIV programme had undergone various changes shortly before the pandemic, the well-established EPI programme was used as a comparator to the HIV programme in selected analyses, with EPI indicators age matched as close as possible to the Early Infant Diagnosis (EID) testing schedule period. The analysed data related to EPI, VTP, HIV testing, diagnosis, treatment initiation and monitoring amongst CLHIV and WLHIV. The NICD DW, which is a subdivision of the National Health Laboratory Service, houses laboratory-related data for all communicable disease testing within the South African public health sector. In lieu of a unique patient identifier, the NICD DW employs a probabilistic algorithm enabling the linkage of multiple HIV tests to an individual, using the name, date of birth, location, and folder number to allocate a unique identifier, thus enabling de-duplicated patient-level data acquisition.^28,29^ Whereas this algorithm has a reported sensitivity of 73% in matching adult HIV test data,^28^ considerable under-linking of infant HIV polymerase chain reaction (PCR) test results has been reported.^30^ To address this, HIV PCR test data were evaluated in specific age bands to reduce the likelihood of double-counting a patient’s results (Online Appendix 1, Table S1).

### Data analysis

The pre-pandemic period (2019/2020) was compared to the pandemic periods (2020/2021, 2021/2022). Visual analyses for CD4 count and HIV VL testing trends (for WLHIV and CLHIV) and HIV diagnosis, loss to follow-up, HIV-related mortality, and EPI coverage (CLHIV only) were performed.

Aggregated NICD DW and DHIS data were exported to an Excel 365 workbook (Microsoft, United States) for analysis. Exported NICD DW data: (1) HIV PCR testing at birth, age 10 weeks and 6 months (total numbers and positive test results); (2) CD4 and HIV VL values among WLHIV and CLHIV. Exported DHIS data: (1) Number of births (total live births and births to WLHIV); (2) Management and outcomes among CLHIV (ART initiation, loss to follow-up, deaths); (3) Public sector EPI-related data (number of administered vaccinations: Bacillus Calmette-Guerin [BCG] at birth; Diphtheria, Tetanus, acellular Pertussis, inactivated Polio, Haemophilus influenzae type b and Hepatitis B virus [DTaP-IPV-Hib-HBV-1 & -4] at 6 weeks and 18 months; Measles-1 at 6 months). DTaP-IPV-Hib-HBV-1 data was the age-aligned EPI comparator for the 10-week HIV PCR test, as 10-week DTaP-IPV-Hib-HBV-2 vaccination data were incomplete.

The calculations and definitions utilised in the study are referenced in Online Appendix 1, Table S1.

### Ethical considerations

Ethical approval for this study was obtained from the Faculty of Health Sciences at the University of Pretoria (reference number 822/2020), the Research Ethics committee of Sefako Makgatho Health Sciences University (SMUREC/M/54/2021), and the University of the Witwatersrand (M210752). Permission was also obtained from the Tshwane District Research Committee. The National Health Research Database number was GP_202011_076. Consent was not required as anonymised and routinely collected data were analysed.

## Results

### Care of women living with HIV

**FIGURE 1:**
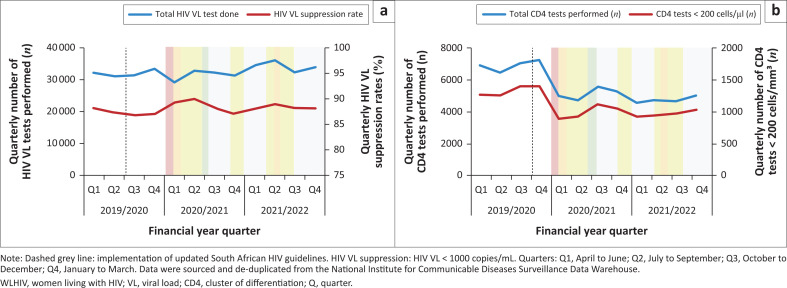
Routine HIV disease monitoring among women living with HIV (15–45 years) per quarter from April 2019 to March 2022 showing (a) HIV VL monitoring among WLHIV, and (b) CD4 count monitoring amongst WLHIV.

Correlating with the period of more restrictive COVID-19 lockdown levels, the year-on-year total HIV VL tests performed among WLHIV declined (*n* = 127 875 [2019/2020]; *n* = 125 444, −2% [2020/2021]), but surpassed pre-pandemic levels in the following FY (*n* = 136 561, +9% [2021/2022]). The annual VL suppression rates remained constant over the study period (87% − 89%) (Figure 1a). There was a decline in both absolute number of CD4 testing (*n* = 27 734 [2019/2020]; *n* = 20 689, −25% [2020/2021]; *n* = 19 082, −7% [2021/2022]) and absolute number of CD4 test results < 200 cells/mL. The proportion of CD4 test results < 200 cells/mL, however, remained constant (*n* = 5342 [19%] [2019/2020]; *n* = 4003 [19%], −25% [2020/2021]; *n* = 3899 [20%], −3% [2021/2022]) (Figure 1b).

### Paediatric HIV diagnosis

Year-on-year total number of deliveries to all women and live births to WLHIV increased initially, but declined to below pre-pandemic levels in the following FY (total number of deliveries: *n* = 59 182 [2019/2020]; *n* = 59 965, +1% [2020/2021]; *n* = 57 736, −4% [2021/2022]; and live births to WLHIV: *n* = 12 942 [2019/2020]; *n* = 13 185, +2% [2020/2021]; *n* = 12 104, −8% [2021/2022]) (Online Appendix 1, Figure S2).

Both birth and 10-week HIV PCR testing coverage remained constant in 2020/2021 but increased in 2021/2022 (testing coverage: birth HIV PCR test: 2019/2020, 101%; 2020/2021, 103%; 2021/2022, 110.4%; and 10-week HIV PCR test: 2019/2020, 66%; 2020/2021, 66%; 2021/2022, 74%).

**FIGURE 2:**
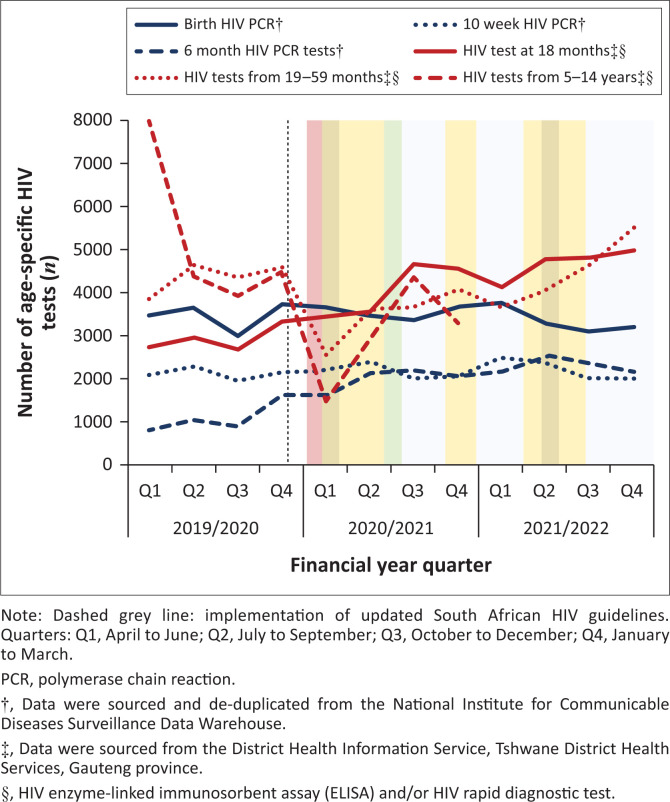
Number of age-specific paediatric HIV tests performed per year and year-on-year percentage change from April 2019 to March 2022.

After the introduction of the 6-month HIV PCR and 18-month universal HIV antibody tests in the 2019 HIV Guidelines, there was a sustained increase in the total number of tests performed (year-on-year change [%]: 6-month HIV PCR: 2019/2020 and 2020/2021, +83%; 2020/2021 and 2021/2022, +15%; 18-month universal HIV test: 2019/2020 and 2020/2021, +38%; 2020/2021 and 2021/2022, +16%). HIV testing, as reported through DHIS, declined among children aged 19–59 months in 2020/2021 but exceeded pre-pandemic numbers the following year (year-on-year change [%]: 2019/2020 and 2020/2021, −20%; 2020/2021 and 2021/2022, +29%). Testing among 5–14-year-olds also declined in 2020/2021, with no data were available for 2021/2022 (year-on-year change [%]: 2019/2020 and 2020/2021, −42%) (Figure 2).

### Care of children living with HIV

The overall birth HIV PCR positivity rate was 0.95% (0.89% – 0.99%) (Table 1). Despite an increase in the number of tests performed in children < 5 years according to NICD and DHIS (Figure 2), the number of positive test results and test positivity declined. In children aged 5–14 years, positive test results also decreased. The HIV EID case rate (cases/100 000 live births) was 485 in 2019/2020, 410 in 2020/2021, and 454 in 2021/2022 (Table 1).

**TABLE 1:** Age-specific HIV positive results and positivity rates performed per year, and year-on-year percentage change from April 2019 to March 2022.

Indicator	Period	Value	Change year-on-year (%)	HIV test positivity rate (%)§
Number of infants with positive HIV PCR tests at birth†	2019/2020	138	-	0.99
	2020/2021	127	−8	0.89
	2021/2022	129	+2	0.97
Number of infants with positive HIV PCR test at 10 weeks of age†	2019/2020	73	-	0.86
	2020/2021	60	−18	0.69
	2021/2022	66	+10	0.74
Number of infants with positive HIV PCR tests at 6 months of age†	2019/2020	76		1.74
	2020/2021	59	−22	0.74
	2021/2022	67	+14	0.73
Number of children with positive HIV tests at 18 months of age‡¶	2019/2020	128	-	1.09
	2020/2021	111	−13	0.68
	2021/2022	126	14	0.67
Number of children with positive HIV tests at 19–59 months of age‡¶	2019/2020	169	-	0.97
	2020/2021	145	−14	1.04
	2021/2022	148	+2	0.83
Number of children with positive HIV tests at 5–14 years old‡¶	2019/2020	353	-	1.69
	2020/2021	309	−12	2.57
	2021/2022	331	+7	0††

PCR, polymerase chain reaction.

† , Data was sourced and de-duplicated from the National Institute for Communicable Diseases Surveillance Data Warehouse.

‡ , Data was sourced from the District Health Information Service, Tshwane District Health Services, Gauteng province.

§ , Calculation, HIV test positivity rate (%): numerator, number of first positive HIV PCR results; denominator, total number of HIV PCR tests performed.

¶ , HIV enzyme-linked immunosorbent assay (ELISA) and/or HIV rapid diagnostic test.

†† , Data incomplete.

**FIGURE 3:**
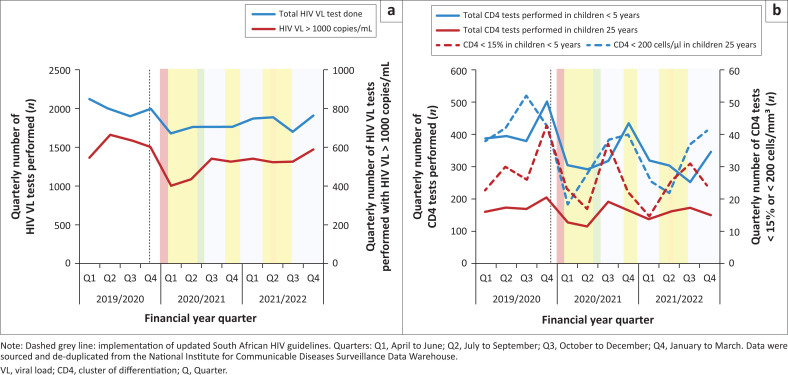
Routine HIV monitoring among children living with HIV per quarter from April 2019 to March 2022. (a) Routine HIV VL tests performed among children living with HIV per quarter from April 2019 to March 2022, (b) Routine CD4 tests performed among children living with HIV per quarter from April 2019 to March 2022.

In 2019/2020, 1692 children were newly initiated on ART, initially declining in 2020/2021 but improving to near pre-pandemic levels in 2021/2022. HIV VL testing among CLHIV also declined in the first year of the pandemic, but by the second year, VL testing levels returned to near pre-pandemic levels. The mean HIV VL suppression rate among CLHIV was 69% in 2019/2020, 73% in 2020/2021, and 70% in 2021/2022 (Figure 3).

The total number of CD4 tests performed in CLHIV declined (2019/2020: *n* = 2370; 2020/2021: *n* = 1953, −18%; 2021/2022: *n* = 1843, −6%) (Figure 3). The absolute number of CD4 test results classified as severe immunosuppression declined, but the percentage remained constant (2019/2020: *n* = 279, 13%); 2020/2021: *n* = 223, 11%); 2021/2022: *n* = 221, 12%) (Figure 3). Cumulative loss to follow-up numbers in CLHIV on the ART programme declined (Table 2) Although the annual cumulative deaths data were incomplete, 12 deaths were recorded in 2019/2020, 10 in 2020/2021, and 19 in 2021/2022.

**TABLE 2:** Yearly outcomes of children (≤ 15 years) living with HIV and year-on-year percentage change from April 2019 to March 2022.

Indicator	Period	Value	% change from preceding year
Number of CLHIV newly initiated on ART	2019/2020	1692	-
	2020/2021	1576	−7
	2021/2022	1663	+5
Number of CLHIV lost to follow-up	2019/2020	373	-
	2020/2021	321	−14
	2021/2022	249	−22

Note: Data were sourced from the District Health Information Service, Tshwane District Health Services, Gauteng province.

ART, Antiretroviral Therapy; CLHIV, Children living with HIV.

### Expanded Program on Immunisation

In 2020/2021, except for BCG, mean monthly vaccine administration volumes at 10 weeks decreased by 9%, and by 11% at both 6 months and 18 months (Table 3). The greatest percentage decrease occurred in April 2020, corresponding with the most restrictive lockdown levels. In 2021/2022, mean monthly vaccine administration rose back to near pre-pandemic levels (Table 3).

**TABLE 3:** Number of vaccinations administered according to select month time-points and year-on-year percentage change from April 2019 to March 2022.

Vaccination	Period (month time-point)	April		July		October		January		Mean	
		Value (*n*)	Change from preceding year (%)	Value (*n*)	Change from preceding year (%)	Value (*n*)	Change from preceding year (%)	Value (*n*)	Change from preceding year (%)	Value (*n*)	Change from preceding year (%)
BCG dose	2019/2020	4536	-	4731	-	4185	-	4638	-	4523	-
	2020/2021	4710	+4	4381	−7	4715	+13	4255	−8	4515	0
	2021/2022	4815	+2	4698	+7	4124	−13	4334	+2	4493	0
DTaP-IPV-Hib-HBV-1 dose	2019/2020	3772	-	4366	-	4062	-	4145	4	086	-
	2020/2021	3249	−14	4087	−6	3947	−3	3670	−11	3738	−9
	2021/2022	3954	+22	3860	−6	3786	−4	3748	+2	3837	+3
Measles-1 dose	2019/2020	3265	-	3889	-	3863	-	3838	-	3714	-
	2020/2021	2355	−28	3605	−7	3825	−1	3418	−11	3301	−11
	2021/2022	3388	+44	3681	+2	4015	+5	3791	+11	3719	+13
DTaP-IPV-Hib-HBV-4 dose	2019/2020	2167	-	2897	-	2819	-	2647	-	2633	-
	2020/2021	1474	−32	2475	−15	2633	−7	2361	−11	2236	−15
	2021/2022	2138	+45	2673	+8	3240	+23	2785	+18	2709	+21

Note: Data were sourced from the District Health Information Service, Tshwane District Health Services, Gauteng province.

BCG, Bacillus Calmette-Guerin; DTaP-IPV-Hib-HBV, Diphtheria, Tetanus, acellular Pertussis, inactivated Polio, Haemophilus influenzae type b and Hepatitis B virus.

Estimated vaccination numbers in infants exposed to HIV and HIV PCR testing comparisons revealed birth HIV PCR testing consistently exceeded DHIS-reported BCG vaccine administration numbers (> 100%); integration of DTaP-IPV-Hib-HBV-1 and Measles-1 administration improved with the age-aligned HIV PCR test (Table 4).

**TABLE 4:** Integration of HIV and vaccination services in infants born to mothers living with HIV: The estimated number of vaccinations administered to infants exposed to HIV compared to the actual number of HIV PCR tests performed from April 2019 to March 2022.

Age	EPI and EID service indicators	Year		
		2019/2020	2020/2021	2021/2022
Birth	HIV PCR†	13 189	13 624	12 514
	BCG dose‡§	11 808	12 635	11 204
	Service integration (%)	112	108	112
10 weeks	HIV PCR†	8498	8657	8919
	DTaP-IPV-Hib-HBV-1 dose‡§	10 133	10 107	9548
	Service integration (%)	84	86	94
6 months	HIV PCR†	4359	7989	9177
	Measles-1 dose‡§	9259	9206	9274
	Service integration (%)	47	87	99

BCG, Bacillus Calmette-Guerin; PCR, polymerase chain reaction; DTaP-IPV-Hib-HBV, Diphtheria, Tetanus, acellular Pertussis, inactivated Polio, Haemophilus influenzae type b and Hepatitis B virus; EPI, Expanded Program on Immunisation; EID, Early Infant Diagnosis; WLHIV, women living with HIV.

† , Data was sourced and de-duplicated from the National Institute for Communicable Diseases Surveillance Data Warehouse.

‡ , Data was sourced from the District Health Information Service, Tshwane District Health Services, Gauteng province.

§ , Calculated estimated number of vaccinations administered to infants exposed to HIV: Monthly estimated total population immunisation coverage: Numerator: Total monthly immunisations administered; Denominator: mean monthly live births. Calculated estimated number of vaccinations administered to infants exposed to HIV: Multiplicand: mean monthly live births to WLHIV; Multiplier: Monthly estimated total population immunisation coverage.

## Discussion

Amidst the unanticipated COVID-19 pandemic, paediatric HIV care rendered by public health facilities within the Tshwane District demonstrated some resilience, notwithstanding the challenges posed by the planned implementation of the updated 2019 HIV guideline. In the analysis of DHIS and NICD data from April 2019 to March 2022, trends emerged in the outpatient care of pregnant women living with HIV and children. Decreases were observed in HIV PCR testing and immunisation rates for infants > 10 weeks of age and HIV test positivity for children < 5 years. Concurrent declines occurred for HIV VL and CD4 testing among pregnant women and children living with HIV. These decreases were most prominent during the most restrictive lockdown levels (Q1 2020/2021). These outpatient services were likely hampered by staff shortages (redeployment to COVID-19 care, illness or bereavement), diminished healthcare capacity, fear of infection, transport availability and restrictive lockdowns, as described in other global settings.^19,31,32^ Birth HIV PCR testing and BCG immunisation rates maintained consistent levels throughout the pandemic period. After the easing of lockdown levels, gradual improvements in most HIV and EPI indicators were seen. By the second pandemic year, most HIV and EPI indicators had recovered to proximate pre-pandemic levels, however, the lack of compensatory increases suggests that some patients may not have returned to care.

In 2020/2021, live births increased, both nationally (+3.6%) and in Tshwane District (+1.3%); possibly linked to reported initial declines in family planning services.^33,34,35^ Disruptions to ante- and postnatal services were, however, variable.^33,36^

In Tshwane District, all maternity units, except for one, continued to render services; these services form part of a well-established obstetric health service. For this analysis, birth HIV PCR testing and BCG coverage were used as a proxy for in-facility postnatal care. Both remained consistent during the pandemic, demonstrating robustness in withstanding the pandemic’s disruptions. Possible reasons for HIV PCR testing exceeding BCG administration can include over-reporting of HIV PCR testing or decreased BCG administration due to stockouts, parental refusal, concurrent tuberculosis preventative therapy (TPT), infant death, or data under-reporting.

HIV VL monitoring among WLHIV was also largely unaffected: the initial decline in VL monitoring corresponded with the most restrictive lockdown levels and recovery to pre-pandemic levels occurred. The HIV viral suppression rates increasing immediately after initial restrictive lockdown levels was similar to findings reported from Italy and Uganda.^37,38^ One postulate is that historically virologically unsuppressed women may have disproportionately disengaged from HIV services or no longer had HIV VL testing performed after the initial restrictive lockdown period. CD4 monitoring remained low as routine testing was no longer part of the guidelines.

Early in the pandemic, global and regional modelling data showed a potential increase in vertical HIV transmission due to lower HIV testing and disrupted prevention of vertical transmission services.^36,39^ From the Tshwane District data this was not evident, with birth and 10-week HIV PCR positivity remaining constant. The updated HIV guidelines saw the implementation of additional 6-month HIV PCR test and universal 18-month testing.

The 6-month HIV PCR test numbers experienced a notable increase. The expected increase in case finding at 6 months did not occur. The expanded 6-month HIV PCR testing and associated decreased test positivity may suggest improvements in VTP due to increased use of dolutegravir-based ART regimens. Conversely, the impact of infant exposure to dolutegravir on the EID assay’s diagnostic sensitivity has yet to be determined. An increase in loss of HIV-detection and associated increase in false-negative PCR results may occur.^40^ To mitigate this, universal 18-month and post-breastfeeding HIV testing uptake should be improved.

Prior to the introduction of the universal 18-month HIV test, only infants exposed to HIV received an HIV test at age 18 months. Universal testing at 18-months aimed to provide a means of diagnosing and linking to care all postnatally infected children. Unfortunately, implementation of this test was found to be poor. The decline in testing among children aged 19 months – 14 years in 2020/2021 may reflect the reduced healthcare-seeking behaviour seen during the pandemic, as HIV testing in this age group would mostly be performed in children accessing acute care and less so for routine well-child visits.

HIV diagnosis and treatment initiation in CLHIV were adversely affected in 2020/2021. Although recovery was observed in 2021/2022, numbers remained below pre-pandemic levels, thus impacting progress towards the updated 2020 UNAIDS goal of 95-95-95.^41^ Compensatory rebounds in diagnosis and treatment initiation should have occurred as lockdown levels eased and access to healthcare improved. This did not occur, meaning that there remained a portion of children who were missed, or who disengaged from care. They need to be brought back into the care environment.

Retaining CLHIV in life-long care is challenging. The practical difficulties posed by the pandemic meant that those who were either newly engaged in care, or historically virologically unsuppressed or immunosuppressed were unlikely to overcome these difficulties.^42^ The reduced HIV VL and CD4 testing numbers but increased HIV VL suppression rates and sustained proportion of severely immunosuppressed CLHIV illustrates that this group of children may have been more likely to disengage from care and forgo routine HIV monitoring. This creates a false impression of improved HIV care. Active client identification and tracking, and a client-centred approach are important strategies to reduce disengagement and improve linkage-to-care and clinical outcomes.^43,44^ Loss-to-follow-up and HIV-related mortality numbers declined among children in Tshwane District. However, further research is warranted to assess whether this is truly reflective or linked to inadequate pandemic-era data collection.

Healthcare access for children was compromised globally during the COVID-19 pandemic, highlighted by the various global immunisation programme disruptions.^19,34,45^ In South Africa, compared to the previous year, the number of children fully immunised at age 12 months declined in 2020/2021 (−4.3%).^34^ In 2020/2021, DTaP-IPV-Hib-HBV-1 and measles vaccinations declined between 9% and 15% in Tshwane District. Following advocacy by various stakeholders to restore the EPI programme coverage, programme reprioritisation occurred and vaccination coverage in Tshwane District improved.^15,17,19,34,45^ These improvements culminated in figures equalling or even surpassing (in the case of DTaP-IPV-Hib-HBV-4) pre-pandemic numbers. Measles-1 immunisation coverage within Tshwane District dropped to 70% in 2020/2021 (47% – 81%) but rebounded to 77% (60% – 83%) in 2021/2022, in line with global trends.^45^ Respiratory infections, such as measles, have a high basic reproductive rate (R_0_); thus, to ensure infection control, populations require very high levels of protection, either directly through vaccinations or natural infections. To maintain this herd immunity, measles requires vaccination coverage of at least 92% – 94%.^46^ Despite the rebound in the coverage, it was still below the target, and a cohort of unvaccinated at-risk children remain who should be targeted for catch-up immunisation activities. Consequently, South Africa has experienced a measles outbreak which began in October 2022. As of June 2023, 39 cases were reported in Tshwane District, comprising 3.6% of national reported cases.^18^

Finally, despite the improved integration of immunisation and EID services at the 6-month primary healthcare well-baby visit, service integration at the 10-week well-baby visit remains sub-optimal as HIV testing remained consistently below the estimated number of vaccinations administered. Paediatric outpatient service integration needs to be strengthened. By accurately documenting the maternal HIV status in the infant’s clinical records and monitoring this cohort using electronic tools, for example, infants exposed to HIV, presenting for routine vaccinations, can be identified and HIV testing offered. Crucially, the early identification and diagnosis of infants living with HIV allows for prompt linkage-to-care, ART initiation and, ultimately, mortality reduction.^47^

### Limitations

Our study has limitations. Interpretation of DHIS data relies upon the preciseness of the data collected: accuracy can be compromised during collection or collation processes. Due to the strain of the pandemic, data collection may have been less meticulous, resulting in either missing, inaccurate or under-reported data. There are also limitations with the NICD data, specifically potential under-linking of the probabilistic record-linking algorithm on account of patient demographic details being incorrectly recorded and no alternative unique patient health-system data identifier available. This may have resulted in over-reporting of the number of infants and children diagnosed by HIV PCR testing as well as VL and CD4 tested numbers.^28,47,48^ EPI coverage data are unable to accurately determine the cohort of infants exposed to HIV attending immunisation services and calculations used in this study are a proxy at best. Cohort monitoring was not done; therefore, EID coverage data were unable to take patient movement across borders into consideration, making it difficult to accurately describe UNAIDS cascades of care.

## Conclusion

The HIV and EPI programmes, in this large urban district in South Africa, were initially adversely impacted during the COVID-19 pandemic. Weaknesses within the healthcare system were exacerbated, leading to reduced immunisation coverage, HIV diagnosis and management among older children, and women and children living with HIV disengaging from care. However, as the pandemic progressed, most indicators returned to proximate pre-pandemic levels. Despite recovering, the pandemic served as a clarion call for healthcare programmes to be strengthened, futureproofing the healthcare system against the occurrence of potential disasters of various kinds and magnitudes. Attaining the stipulated HIV and EPI goals will require bringing children disengaged by the COVID-19 pandemic back into the healthcare system.
